# Immunogenicity and safety of xenogeneic vascular endothelial growth factor receptor-2 DNA vaccination in mice and dogs

**DOI:** 10.18632/oncotarget.7265

**Published:** 2016-02-08

**Authors:** Sofie Denies, Laetitia Cicchelero, Ingeborgh Polis, Niek N. Sanders

**Affiliations:** ^1^ Laboratory of Gene Therapy, Department of Nutrition, Genetics and Ethology, Faculty of Veterinary Medicine, Ghent University, Merelbeke, Belgium; ^2^ Small Animal Hospital, Department of Medicine and Clinical Biology of Small Animals, Faculty of Veterinary Medicine, Ghent University, Merelbeke, Belgium

**Keywords:** immunotherapy, DNA vaccine, electroporation, VEGFR-2, cancer, Immunology and Microbiology Section, Immune response, Immunity

## Abstract

Vascular endothelial growth factor receptor-2 (VEGFR-2) is an attractive target in oncology due to its crucial role in angiogenesis. In this study a DNA vaccine coding for human VEGFR-2 was evaluated in healthy mice and dogs, administered by intradermal injection and electroporation. In mice, three doses and vaccination schedules were evaluated. Cellular immune responses were measured by intracellular IFN-gamma staining and a cytotoxicity assay and antibodies by ELISA. Safety was assessed by measuring regulatory T cells and myeloid derived suppressor cells and a wound healing assay. The vaccine was subsequently evaluated in dogs, which were vaccinated three times with 100μg. Cellular immune responses were measured by intracellular IFN-gamma staining and antibodies by a flow cytometric assay. In mice, maximal cellular responses were observed after two vaccinations with 5μg. Humoral responses continued to increase with higher dose and number of vaccinations. No abnormalities in the measured safety parameters were observed. The vaccine was also capable of eliciting a cellular and humoral immune response in dogs. No adverse effects were observed, but tolerability of the electroporation was poor. This study will facilitate the evaluation of the vaccine in tumor bearing animals, ranging from rodent models to dogs with spontaneous tumors.

## INTRODUCTION

It is well-known that the immune system is capable of recognizing tumor cells and to establish a specific long-term antitumor response. Therefore, vaccination against tumor antigens holds great promises in the treatment for cancer [1]. However, due to complex interactions between tumor cells and the immune system and the problem of tolerance, the translation of the theoretical potential of tumor vaccines into an effective clinical response has been difficult [2].

In healthy individuals, vascular endothelial growth factor-2 (VEGFR-2) is expressed on virtually all endothelial cells as well as on certain other cell types including retinal progenitor cells, megakaryocytes and pancreatic duct cells. In cancer patients, VEGFR-2 is strongly overexpressed on tumoral blood vessels [3]. VEGFR-2 can thus be classified as a ubiquitous tumor antigen. The potential of raising an effective immune response against these tumor antigens without causing auto-immunity can be explained by the level of overexpression in tumors, reaching the threshold for T cell recognition and thus breaking immune tolerance [4]. As it is difficult to raise a strong immune response against self-antigens, xenogeneic vaccination, which involves the use of an antigen of a different species, has been proven to result in more potent immune responses [5].

VEGFR-2 as vaccine target has some important advantages. Tumor cells have grown under the selective pressure of the immune system. Evading immune control is one of the hallmarks of tumors, with down regulation of MHC-1 molecules and resistance to cytotoxic effects of immune cells as two common examples [1]. These immune evasive strategies acquired during tumor growth make tumor cells difficult targets for vaccine strategies. Moreover, because of their genetic instability, tumor antigens in tumor cells are prone to mutations, which leads to escape mutants that further decrease the efficacy of tumor vaccines [1]. Endothelial cells of tumoral blood vessels have not acquired immune evasive strategies and are genetically stable [6]. They are thus more vulnerable to vaccination strategies. Targeting tumor vasculature instead of tumor cells directly has proven its value, for instance by the success of bevacizumab [3].

Although targeting VEGFR-2 is primarily an anti-angiogenic treatment, certain tumors also express VEGFR-2, serving as an autocrine growth factor for these cells [7]. In these tumor types VEGFR-2 vaccination can also induce a direct antitumor effect. Moreover, VEGFR-2 is expressed on regulatory T cells (Tregs) and myeloid derived suppressor cells (MDSCs) [8, 9]. Targeting these cells via VEGFR-2 vaccination can have a self-reinforcing effect, as these cells suppress anti-tumor immune responses [1].

VEGF/VEGFR targeting has been explored extensively with different treatment modalities, with some of them approved for clinical use [6]. In preclinical studies vaccination against VEGFR-2 has also been explored [10]. Vaccination has some important advantages. It is more specific than small molecule inhibitors and compared to monoclonal antibodies the polyclonal antibody response evoked by vaccination may have a higher antigen neutralizing capacity. Both monoclonal antibodies and small molecule inhibitors have to be administered frequently, which makes vaccination a much more cost efficient therapy [6]. Different vaccine platforms exist. DNA vaccination has the advantage that the antigen is synthetized intracellularly, leading to a robust cellular immune response [11]. Moreover, DNA vaccines often make use of plasmids that contain immune stimulating unmethylated CpG motives [12]. Electroporation mediated delivery of DNA vaccines can further support the development of an immune response because the limited tissue damage caused by electroporation also serves as an adjuvant [13]. Delivery of DNA vaccines via electroporation has also the advantage that the vaccine manufacture is more economical, uniform and suitable for large scale production than when viral or chemical carriers are used for the delivery [14, 15].

In this study we evaluated in healthy mice and dogs the safety and immunogenicity of a xenogeneic VEGFR-2 DNA vaccine. The DNA vaccine was administered as a naked plasmid in combination with needle array electroporation.

## RESULTS

### Effect of vaccine dose and vaccination schedule on the immune response in mice

#### Cellular immune response in mice

**Figure 1:**
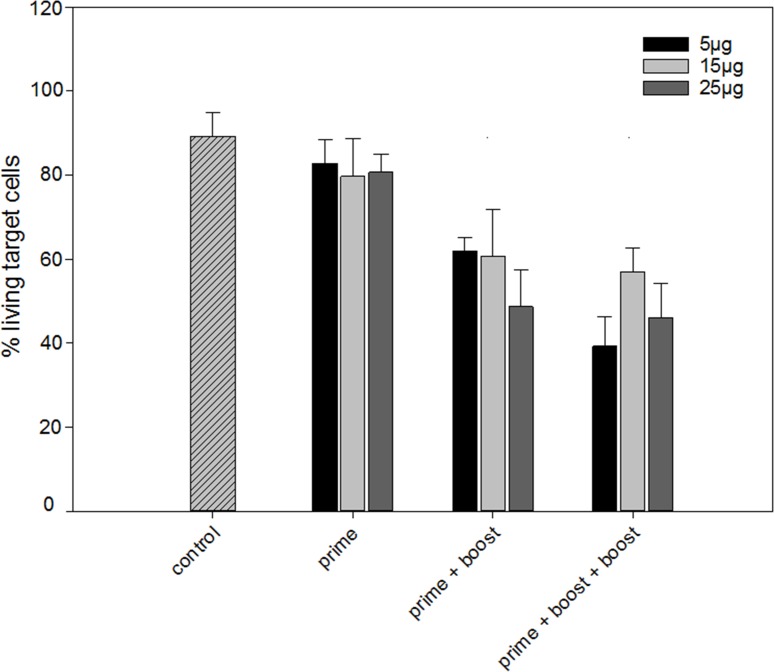
Cytotoxic activity of splenocytes isolated from non-vaccinated and vaccinated mice that received one (prime), two (prime + boost), or three (prime + boost + boost) VEGFR-2 DNA vaccinations Three different DNA vaccine doses (5, 15 or 25 μg) were tested. Two weeks after the last vaccination splenocytes were collected and their capacity to lyse VEGFR-2 expressing target cells was measured. (control indicates non-vaccinated mice, bars represent mean, error bars standard error of the mean, *n* = 4).

To study the influence of the VEGFR-2 DNA dose and vaccination schedule on the induction of tumor antigen specific cytotoxic T-lymphocytes we incubated splenocytes with tumor cells expressing human VEGFR-2 at an effector:target ratio of 20:1. Splenocytes from vaccinated mice demonstrated significant higher cytotoxicity than those from control mice (*p* = 0.015) without a significant effect of the DNA vaccine dose (Figure 1). When we tested the effect of the different vaccination schedules on the cytotoxic T-lymphocytes we found that one boost is superior to none (*p* = 0.002). Overall, there was no significant difference between three and two vaccinations, although for 5μg a trend towards better response after three vaccinations is visible. After co-incubation with splenocytes from mice vaccinated at least two times there were on average 52% surviving target cells (standard deviation (SD) 14%), compared to 89% (SD 13%) when co-incubated with splenocytes from non-vaccinated mice. There was no difference in response towards tumor cells not expressing human VEGFR-2, demonstrating a specific response towards the antigen.

**Figure 2:**
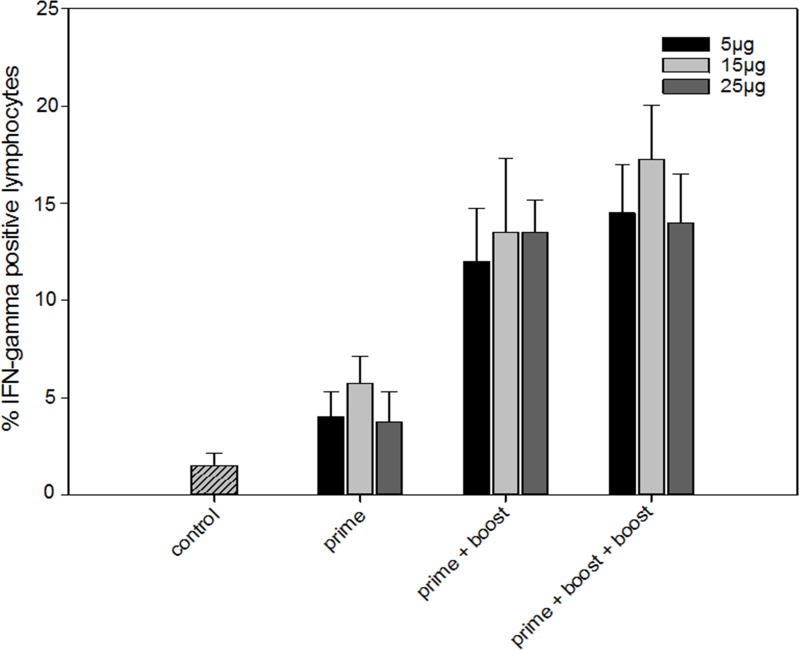
Interferon-gamma response of splenocytes isolated from non-vaccinated and vaccinated mice that received one (prime), two (prime + boost), or three (prime + boost + boost) VEGFR-2 DNA vaccinations Three different DNA vaccine doses (5, 15 or 25 μg) were tested. Two weeks after the last vaccination splenocytes were collected and stimulated with murine VEGFR-2 protein. Following stimulation the percentage of IFN-gamma positive splenocytes was measured by flow cytometry after intracellular cytokine staining. (control indicates non-vaccinated mice, bars represent mean, error bars standard error of the mean).

Analysis of IFN-gamma secretion leads to the same conclusions (Figure 2). After overnight incubation with a murine VEGFR-2 protein there were on average 14% (SD 5%) IFN-gamma positive lymphocytes in the splenocytes of mice that received at least two vaccinations, compared to 1% (SD 1%) in splenocytes from non-vaccinated mice. Again, no difference in IFN-gamma secretion was observed in non-stimulated splenocytes.

#### Humoral immune response in mice

The impact of the VEGFR-2 DNA dose and vaccination schedule on the induction of antibodies is summarized in Table 1. Both DNA vaccine dose and vaccination schedule had a significant impact on antibody titer (*p* < 0.001 and *p* = 0.003 respectively). Antibody responses were undetectable in mice vaccinated with the lowest dose (5μg) and for all doses after only one vaccination. The highest antibody titers were present in serum from mice vaccinated three times with 25μg, reaching an antibody titer of 800 in this group.

**Table 1:** Effect of VEGFR-2 DNA vaccine dose and vaccination schedule on VEGFR-2 antibody titers All four mice within each group had the same antibody titer

	Prime	Prime + boost	Prime + boost + boost
5μg	<4	<4	<4
15μg	<4	4	100
25μg	<4	400	800

### Safety in mice

Vaccination with DNA encoding human VEGFR-2 had no effect on normal levels of Tregs (2.38 ± 0.49 in non-vaccinated mice versus 2.57 ± 0.51% in vaccinated mice) or MDSCs in the spleen of healthy mice (1.68 ± 0.35% in non-vaccinated mice versus 1.70 ± 0.32% in vaccinated mice). There was also no effect on the mean time until wound closure (5 days for both vaccinated and non-vaccinated mice, SD 1 day).

### Immunogenicity in dogs

#### Cellular immune response in dogs

**Figure 3:**
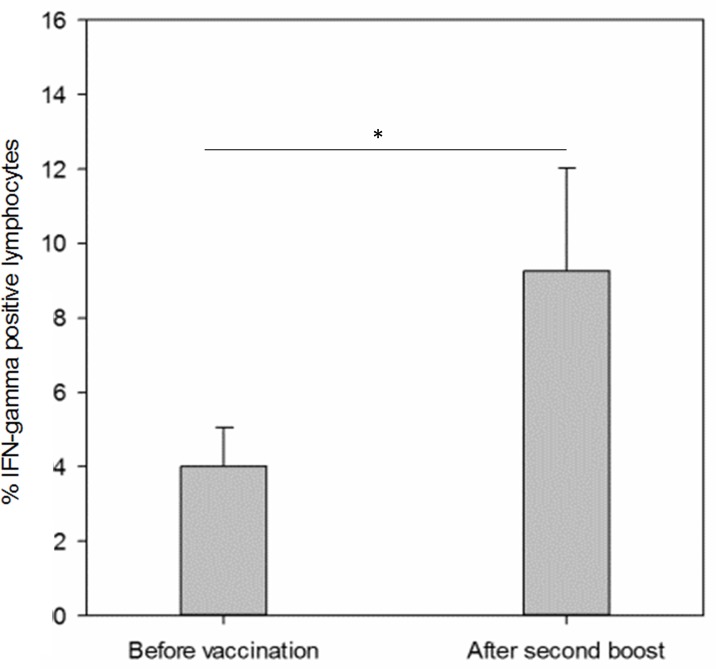
Interferon-gamma response of lymphocytes isolated from dogs before vaccination and after a second boost with the DNA vaccine Two weeks after the second boost lymphocytes were collected and stimulated with human VEGFR-2. Following stimulating the percentage of IFN-gamma positive lymphocytes was measured by flow cytometry after intracellular cytokine staining. (Bars represent mean, error bars standard error of the mean, *p < 0.05).

Before the start of the vaccination and two weeks after each vaccination peripheral blood mononuclear cells (PBMCs) were collected from the dogs and stimulated with human VEGFR-2. Subsequently, the percentage of IFN-gamma positive PBMCs was measured by flow cytometry after intracellular staining. After the first and second vaccination we could not detect a significant IFN-gamma response compared to baseline (data not shown). However, after the third vaccination (i.e. second boost) the percentage of IFN-gamma positive lymphocytes were significantly increased with an average of 9% of IFN-gamma positive lymphocytes (SD 0.05%) compared to 4% (SD 0.02%) at baseline (*p* = 0.04) (Figure 3). No change in IFN-gamma secretion in non-stimulated PBMCs compared to baseline was present at any time point.

We also tried to measure the cytotoxic T cell response after VEGFR-2 DNA vaccination of dogs by co-incubating PBMCs with a canine hemangiosarcoma (HSA) cell line. However, at baseline (i.e. before vaccination) there was already a considerable cytotoxic response of isolated PBMCs towards the canine HSA cell line, with only 26% (SD 9%) HSA cells surviving the co-incubation. No increase in this cytotoxic response was observed after the last vaccination (data not shown). This demonstrates that the allogeneic nature of the target cells already triggered a significant cytotoxic response from naïve PBMCs, as previously reported for human PBMCs [18]. It is hypothesized that this response is already at maximal capacity at baseline explaining no increase after vaccination.

#### Humoral Immune response in dogs

**Figure 4:**
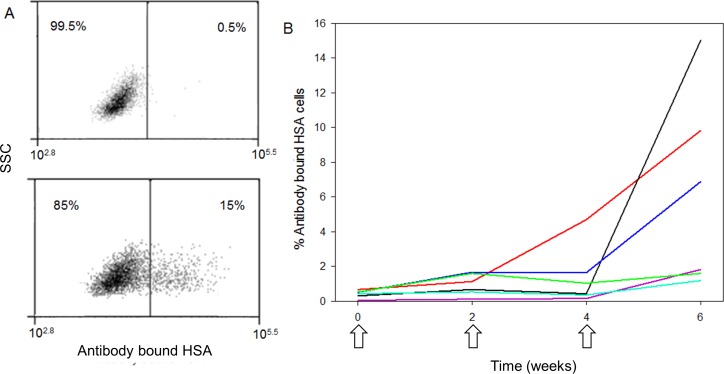
Humoral immune response in dogs Left (**A**): Flow cytometric plots illustrating the evaluation of antibody response by the amount of VEGFR-2 expressing target cells bound with antibodies present in serum via a secondary anti-dog IgG antibody (upper: at baseline, lower: after the last vaccination of the same dog). Right (**B**): time course of the antibody response for individual dogs, arrows indicate vaccination moments and each colored line represents an individual dog. (HSA = hemangiosarcoma; SSC = side scatter)

In vaccinated dogs antibody levels against VEGFR-2 had significantly increased (relative to baseline levels) after the third vaccination (*p* = 0.03). When looking at individual dogs, four out of six dogs had a clear increase in antibodies after the second boost (Figure 4).

### Tolerability in dogs

The delivery of the very short electric pulses caused in all dogs a startle response characterized by withdraw movements, vocalization and stress-induced anal sac excretion. This only occurred during electroporation, which lasted less than one second. Immediately after electroporation, dogs exhibited normal behavior and no reaction when touching electroporation site could be elicited. With the Agilepulse device, actual resistance of the tissue during electroporation is measured and saved. Hence it could be checked whether the electrodes remained in place during the withdraw movements. Based on the resistance data we could conclude that the withdraw movements did not affect any of the electroporations. This is most likely because the total duration of pulses is less than a second. Twenty-four hours after electroporation, the electroporation site was red and swollen, which evolved over two weeks to an epidermal crust. All physical parameters (heart rate, respiration rate, temperature, blood pressure) remained stable during the vaccination period.

## DISCUSSION

This study demonstrates that a human VEGFR-2 DNA vaccine, administered in combination with electroporation, is capable of inducing a cellular and humoral immune response in mice and dogs. The dose escalation study in mice demonstrated that it was easier to elicit a cellular immune response than a humoral immune response. This is in agreement with other studies with DNA vaccines and can be explained by the limited amounts of proteins released after DNA vaccination, which is insufficient for a robust antibody response [19]. A very small amount of antigen when presented within an antigen presenting cell, either directly by transfection or indirectly via cross-presentation, is sufficient to prime strong cytotoxic T cell responses [19]. In our mice study a cellular immune response was already observed after prime-boost vaccination with 5μg pDNA, while higher DNA vaccine doses and at least two vaccinations were needed to elicit a humoral immune response. VEGFR-2 is a transmembrane protein, and thus accessible for both arms of the immune system [3]. Indeed, it has been reported that both a humoral and a cellular response against VEGFR-2 can convey antitumor effects [20]. Further evaluation of the efficacy of our VEGFR-2 DNA vaccine in tumor bearing mice should thus be done with at least 25μg and three vaccinations. Vaccination against VEGFR-2 did not affect wound healing or normal levels of Tregs or MDSCs in healthy mice. Both Tregs and MDSCs significantly upregulate their VEGFR-2 expression in response to tumor secreted cytokines [8, 9]. It is thus possible that in tumor bearing hosts, VEGFR-2 vaccination has a differential effect on the percentage of Tregs and MDSCs compared to healthy individuals.

Dose extrapolation of DNA vaccines from mice to humans or other animals is difficult. A linear extrapolation based on body weight is often used to calculate the dose in other species. From such calculations it is frequently concluded that the doses are too high to be feasible in humans. However, this reasoning is not correct. Firstly, correct allometric translation of drug doses should be done based on body surface area, which results in drug doses 0.08 times less than when based on simple conversion based on body weight [21]. Secondly, allometric scaling of vaccine doses is not relevant, as this is based on pharmacokinetic characteristics that do not influence the interactions between antigen and immune cells after local injection [22]. Many other species specific factors do influence vaccine efficiency, that are too complex to allow for a scaling formula. Therefore, the DNA vaccine dose for dogs in this study (100 μg) was not based on a conversion from the results in mice, but on studies that tested xenogeneic anti-cancer DNA vaccines in dogs delivered with electroporation [23, 24]. The xenogeneic VEGFR-2 DNA vaccine was successful in inducing a cellular and humoral immune response in dogs. A cellular immune response was evident by interferon-gamma secretion after incubation with human VEGFR-2 protein. The immune response against human VEGFR-2 takes more time to develop in dogs compared to mice, with a clear response only after three vaccinations. This timeframe is in agreement with other studies with DNA vaccines in dogs [23, 24]. Our xenogeneic DNA vaccine is based on human VEGFR-2. Human VEGFR-2 shows a higher homology with canine than with murine VEGFR-2 (93% versus 86%). This may explain the higher immunogenicity of our vaccine in mice.

The normal levels of Tregs and MDSCs, wound healing in healthy mice and physiological parameters (heart rate, respiration rate, temperature and blood pressure) in healthy dogs are not influenced by the vaccine. This indicates that no acute side effects occur after administration of our VEGFR-2 DNA vaccine. The tolerability of the electroporation on butorphanol sedated dogs in combination with local anesthesia is poor. As humans treated with the same protocol report good tolerability of electroporation and given the nature of reactions of the dogs, responses are believed to come from startling more than pain [25, 26]. Based on the reaction of the dogs one may consider to use higher levels of sedation or even anesthesia before electroporation. Because of the very short duration of the entire procedure and based on evaluation of experienced veterinarians supervising the experiment this is not ethically imposed, considering discomfort caused by additional manipulations and recovery associated with deep sedation or anesthesia. However, since the safety of the operators is also an aspect that has to be taken into account, higher levels of sedation are in our opinion advised. The inflammation and limited tissue damage observed at the vaccination site illustrates the adjuvant effect of electroporation and did not impact the dogs' welfare in any way.

Different DNA vaccines targeting VEGFR-2 have been evaluated in the past. This wide interest in VEGFR-2 vaccination demonstrates the potential of this approach. The majority of studies with VEGFR-2 DNA vaccines used a viral, bacterial or chemical vector to facilitate the delivery [17, 20, 27-33]. Uniform large scale production is difficult with chemical and biological vectors, making clinical use outside a research context challenging [14, 15]. Biological vectors also raise safety concerns [34]. Vaccination with naked plasmid is evaluated in three research papers [35-37]. Transfection efficiency of naked plasmid however is very low, making this approach far from optimal. However, when naked plasmid is delivered by the aid of a physical gene delivery method it is possible to combine the advantage of industrial suitable vaccine manufacturing with high transfection efficiency [38]. Moreover, physical gene delivery methods lack vector related toxicity issues. Electroporation mediated delivery of a VEGFR-2 DNA vaccine has only been evaluated once in a preclinical setting [39]. However, the vaccine in this study was developed to include only B cell epitopes, excluding cellular immune responses, whereas our vaccine is able to elicit both humoral and cellular immune responses.

As the immunogenicity and safety of this xenogeneic VEGFR-2 DNA vaccine is demonstrated in dogs and mice, the next step is the evaluation of its efficacy in cancer bearing animals. Spontaneously arising tumors in dogs can be a very valuable intermediate model, potentially limiting the now tremendous amount of negative phase I trials in human patients because of the low predictive power of rodent models [40]. Many tumors in dogs share biological, histological and clinical characteristics with their human counterparts and responses to treatment are very similar. Genetic research has revealed that tumors in humans and dogs undergo nearly identical genetic changes. An additional advantage of dogs is their body size, allowing surgical interventions, medical imaging and tissue/blood sampling much like in human patient [41]. Several tumor vaccines have first been evaluated in canine patients, followed by a clinical trial in humans [42-45]. The evaluation of tumor vaccines in clinical trials with dogs can also lead to licensing of the vaccine for veterinary use. The licensing of Oncept, a xenogeneic DNA vaccine encoding human tyrosinase for the treatment of melanoma in dogs has led to its widespread use in veterinary oncology [46]. Similar to humans VEGFR-2 overexpression is confirmed in numerous canine cancer types [47-55]. In conclusion, our data and the many reports on VEGFR-2 will facilitate the evaluation of our xenogeneic VEGFR-2 DNA vaccine in tumor bearing animals, ranging from preliminary rodent models to highly translational models of spontaneous tumors in pet dogs.

## MATERIAL AND METHODS

### Tumor cell lines and VEGFR-2 plasmid

The B16-F10 tumor cell line was a kind gift from Johan Grooten (Department of Biomedical Molecular Biology, Ghent University, Belgium) and was stably transduced with luciferase as described earlier [16]. Briefly, retroviral vectors encoding luciferase were produced in HEK293T cells by calcium phosphate transfection. Virions were harvested after 48 and 72h, filtered and concentrated by ultracentrifugation. Transduction the B16-F10 cells was performed in the presence of 8 μg/ml polybrene and transfection efficiency was evaluated by bioluminescence. The canine hemangiosarcoma (HSA) cell line was a kind gift of Douglas Thamm (College of Veterinary Medicine and Biomedical Sciences, Colorado State University, USA). These cells were grown in Dulbecco's modified Eagle's medium, supplemented with 10% fetal calf serum, 100 mg/ml streptomycin, 100 IU/ml penicillin and 1 mmol/ml L-glutamine (Invitrogen, Carlsbad, CA, USA). The plasmid encoding human VEGFR-2 was purchased form Invivogen (Toulouse, France; pUNO1-hFLK1(mb)). Purification of the plasmids was done with the EndoFree Giga kit, following the instructions of the manufacturer (Qiagen, Valencia, CA, USA).

### DNA vaccine doses and vaccination schedules in mice

**Figure 5:**
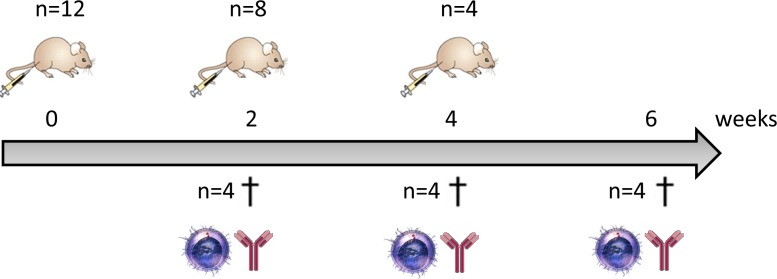
Vaccination schedule in mice Mice were vaccinated with two weeks interval, two weeks after each vaccination four mice were sacrificed to measure cellular and humoral immune response. This vaccination schedule was used to evaluate three different doses of the VEGFR-2 DNA vaccine (5, 15 and 25μg).

The vaccination experiments in mice were approved by the ethical committee of the Faculty of Veterinary Medicine (Ghent University; EC2013/77). Healthy C57BL/6JRj mice of 8 weeks old were used for this optimization study (Janvier Breeding Center, Le Genest St. Isle, France). Three doses (5μg, 15μg and 25μg) and three vaccination schedules (prime, prime-boost, prime-boost-boost) were evaluated with 4 mice in each group (Figure 5). A group of untreated mice served as control. The VEGFR-2 plasmid was injected intradermally in 20μl calcium and magnesium free phosphate buffered saline (PBS; Invitrogen) followed by electroporation with the BTX Agilepulse device (Harvard apparatus, Holliston, MA, USA) using 4 mm gap needle electrodes (2 pulses of 450 V with a pulse duration of 0.05 ms and a 300 ms interval, followed by 8 pulses of 100 V with a pulse duration of 10 ms and a 300 ms interval). Mice were anesthetized with isoflurane during this procedure. The interval between the vaccinations was 2 weeks and mice were euthanized 2 weeks after the last vaccination.

### Assessing cellular immune response in vaccinated mice

Splenocytes were isolated two weeks after the last vaccination. To assess cytotoxic response to cells expressing the target, splenocytes were co-incubated with bioluminescent B16-F10 melanoma cells that were *in vitro electroporated* with a plasmid encoding human VEGFR-2. Electroporation was performed in 2mm cuvettes with the BTX ECM 830 device (Harvard apparatus, Holliston, MA, USA). Two pulses of 140 V with a pulse duration of 5 ms and 100 ms interval were used. As a control for specificity to the VEGFR-2 target, B16-F10 melanoma cells that were mock electroporated were also included. Absence of natural VEGFR-2 expression on the B16-F10 cells and induction of expression after electroporation were assessed by flow cytometry with a rabbit antibody that binds VEGFR-2 and an Alexafluor-688 conjugated secondary goat anti-rabbit antibody (Abcam, Cambrige, UK). After transfection, human VEGFR-2 was expressed on 78% of the tumor cells. To measure the tumor killing capacity of lymphocytes from vaccinated and non-vaccinated mice we incubated 2×10^5^ splenocytes with 10^4^ B16-F10 cells. After 24 hours the bioluminescent signal, which is related to the number of living cells, was measured with an IVIS lumina II (Perkin-Elmer, Zaventem, Belgium).

Additionally, the IFN-gamma response was also measured. In more detail, 10^6^ splenocytes were stimulated overnight with 2μg of murine VEGFR-2 protein (Bio-Connect, Te Huissen, The Netherlands). Subsequently, brefeldin A was added and after 4 hours the cells were incubated overnight in fixation buffer (ImTec Diagnostics, Antwerp, Belgium). The following day the cells were permeabilized and the intracellular staining of IFN-gamma was performed with an APC-conjugated anti-IFN-gamma antibody (Imtec Diagnostics). The cells were analyzed with an Accuri C6 flow cytometer (BD Biosciences, Erembodegem, Belgium) and events in the permeabilized lymphocyte gate were selected for analysis. Controls for specificity of the cytokine response were assessed by including non-stimulated splenocytes in the analysis.

### Assessment humoral immune response in vaccinated mice

Blood was collected 2 weeks after the last vaccination of each vaccination schedule by cardiac puncture under terminal anesthesia. Blood was centrifuged (10min, 3000g, Eppendorf centrifuge) and serum was collected and stored at −80°C until analysis. Microtiter plates were coated overnight with 1μg murine VEGFR-2 protein in coating buffer and loaded with serial dilutions of serum in ELISA assay diluent for 2 hours (Imtec Diagnostics). After washing, a HRP-conjugated anti-mouse IgG antibody was added, followed by development with TMB substrate and measuring absorbance with a microplate reader at 450nm (ImTec Diagnostics). The titer was determined as the limiting dilution with a signal exceeding mean plus twice the standard deviation of the signal from control samples (non-vaccinated mice).

### Assessment of regulatory T cells and MDSCs levels

As VEGFR-2 expression is reported on Tregs and MDSCs, the effect of vaccination on normal levels of these cells was assessed as a measure of safety. Splenocytes were isolated when mice were sacrificed and the percentage of Tregs and MDSCs were measured by flow cytometry. Regulatory T cells were stained with a FITC-conjugated anti-CD25 antibody, an APC-conjugated CD4 antibody and a PE-conjugated anti-FoxP3 antibody after fixation and permeabilization with commercial buffers (Ebioscience, Vienna, Austria). MDSCs were stained with a FITC-conjugated anti-CD11b antibody and a PE-conjugated anti-Gr1 antibody (Ebioscience). The cells were analyzed with an Accuri C6 flow cytometer and events in the lymphocyte gate were selected for analysis. Color compensation was based on fluorescence minus one (FMO) controls.

### Assessment of wound healing in vaccinated mice

To further assess safety of VEGFR-2 vaccination, a wound healing assay was performed. Two circular full-thickness dermal wounds were created between the shoulder blades with a 2-mm punch biopter one week after the last vaccination. These wounds were allowed to heal spontaneously and the days until macroscopic closure of the wound were determined, as described earlier.[17]

### Vaccination of dogs

The vaccination experiments in dogs were approved by the ethical committee of the Faculty of Veterinary Medicine (Ghent University; EC2013/40). Six laboratory Beagle dogs were vaccinated three times with 100μg of human VEGFR-2 plasmid dissolved in 100μl magnesium and calcium free PBS. The vaccine was injected intradermally followed by electroporation with the BTX Agilepulse device (2 pulses of 450 V with a pulse duration of 0.05 ms and a 0.2 ms interval, followed by 8 pulses of 100 V with a pulse duration of 10 ms and a 20 ms interval; Harvard apparatus). Dogs were sedated by intravenous injection of 0.2mg/kg butorphanol (Vetergesic, Alstoe Limited, York, UK). Local anesthesia was obtained by subcutaneous injection of 0.5ml of lidocaine 2% (Xylocaïne, Recipharm Monts, Monts, France) at the vaccination site.

### Assessment of cellular immune response in vaccinated dogs

Blood was collected before each vaccination and two weeks after each vaccination. Blood was collected in EDTA tubes and peripheral blood mononuclear cells (PBMCs) were isolated by centrifugation with Ficoll-Paque 1.007g/ml (Invitrogen). The canine HSA cell line was used to assess a cytotoxic response against VEGFR-2 expressing cells. Expression of VEGFR-2 by these cells was confirmed with an anti-VEGFR-2 antibody and a secondary Alexa fluor 688-conjugated goat anti-rabbit antibody (both from Abcam). Subsequently, the canine HSA cells were in vitro transfected with eGFP via electroporation with the BTX device. A cytotoxicity assay was performed by incubating 2×10^5^ PBMCs with 10^4^ eGFP transfected HSA cells and 24 hours later the number of eGFP expressing cells was analyzed by flow cytometry as a measure of PBMCs mediated killing of VEGFR-2 positive cells. PBMCs obtained before vaccination served as controls.

Additionally, the IFN-gamma response was measured after overnight incubation of 10^6^ PBMCs with 2μg human VEGFR-2 protein, as a recombinant canine VEGFR-2 protein is not available (Acro Biosystems, London, UK). Subsequently, brefeldin A was added followed after 4 hours by overnight incubation in fixation buffer (Ebioscience). The next day, PBMCs were stained with a FITC-conjugated anti-IFN-gamma antibody after permeabilization with a commercial buffer (Life Technologies, Ghent, Belgium). The cells were analyzed with an Accuri C6 flow cytometer and events in the permeabilized lymphocyte gate were selected for analysis. Controls for specificity of the cytokine response were assessed by including non-stimulated PBMCs in the analysis.

### Assessment of the humoral immune response in vaccinated dogs

Plasma was collected two weeks after each vaccination. HSA tumor cells were washed and resuspended in 50μl of diluted plasma (1/8 dilution in FACS buffer, Imtec Diagnostics). Cells were incubated for one hour at 37°C and 5% CO_2_. Subsequently the cells were washed and stained with APC-conjugated anti-dog IgG antibodies (Imtec Diagnostics) and the number of positive cells was analyzed via flow cytometry. Control for specificity of staining included HSA cells that were not incubated with plasma and stained with the secondary antibodies.

### Assessment of tolerability and safety of the vaccination in dogs

As this is the first time that the Agilepulse was used for electroporation of dogs, an important outcome measurement was also the tolerability to the electroporation. Acute stress and pain signals during the electroporation as well as the aspect and sensitivity of the vaccination site the days after electroporation were monitored. A thorough physical examination including Doppler blood pressure monitoring was performed the week after each vaccination to assess safety of the vaccine.

### Statistics

Data were analyzed with the SPSS software version 19 (IBM, Brussels, Belgium). The effect of DNA vaccine dose and vaccination schedule on the cytotoxicity and IFN-gamma secretion of murine splenocytes were analyzed with a general linear model. The effect of DNA vaccine dose and vaccination schedule on antibody titers in serum of mice were analyzed with an exact linear by linear association test. The effect of vaccination on IFN-gamma secretion of canine PBMCs was analyzed with a repeated measures ANOVA. The effect of vaccination on antibody bound HSA cells were analyzed with the non-parametric Friedman test. For post hoc tests, correction for multiple comparisons was performed with the Tukey method.
